# Protein tyrosine kinase but not protein kinase C inhibition blocks receptor induced alveolar macrophage activation

**DOI:** 10.1155/S0962935193000523

**Published:** 1993

**Authors:** K. Pollock, M. T. Withnall

**Affiliations:** Rhône-Poulenc Rorer Ltd Dagenham Research Centre Rainham Road South Essex Dagenham RM10 7XS UK

## Abstract

The selective enzyme inhibitors genistein and Ro 31-8220 were used to assess the importance of protein tyrosine kinase (PTK) and protein kinase C (PKC), respectively, in N-formyl-methionyl-leucyl-phenylalanine (FMLP) induced generation of superoxide anion and thromboxane B_2_ (TXB_2_) in guinea-pig alveolar macrophages (AM). Genistein (3–100 μM) dose dependently inhibited FMLP (3 nM) induced superoxide generation in non-primed AM and TXB_2_ release in non-primed or in lipopolysaccharide (LPS) (10 ng/ml) primed AM to a level > 80% but had litle effect up to 100 μM on phorbol myristate acetate (PMA) (10 nM) induced superoxide release. Ro 31-8220 inhibited PMA induced superoxide generation (IC_50_ 0.21 ± 0.10 μM) but had no effect on or potentiated (at 3 and 10 μM) FMLP responses in non-primed AM. In contrast, when present during LPS priming as well as during FMLP challenge Ro 31-8220 (10 μM) inhibited primed TXB_2_ release by > 80%. The results indicate that PTK activation is required for the generation of these inflammatory mediators by FMLP in AM. PKC activation appears to be required for LPS priming but not for transducing the FMLP signal; rather, PKC activation may modulate the signal by a negative feedback mechanism.


Mediators of Inflammation 2, 373–377 (1993)

**The selective enzyme inhibitors genistein and Ro 31-8220 were used to assess the importance of protein tyrosine kinase (PTK) and protein kinase C (PKC), respectively, in *N*-formyl-methionyl-leucyl-phenylalanine (FMLP) induced generation of superoxide anion and thromboxane $B_2$ ($TXB_2$) in guinea-pig alveolar macrophages (AM). Genistein (3–100 $\mu$M) dose dependently inhibited FMLP (3 nM) induced superoxide generation in non-primed AM and $TXB_2$ release in non-primed or in lipopolysaccharide (LPS) (10 ng/ml) primed AM to a level > 80% but had litle effect up to 100 $\mu$M on phorbol myristate acetate (PMA) (10 nM) induced superoxide release. Ro 31-8220 inhibited PMA induced superoxide generation ($IC_{50}$ 0.21 $\pm$ 0.10 $\mu$M) but had no effect on or potentiated (at 3 and 10 $\mu$M) FMLP responses in non-primed AM. In contrast, when present during LPS priming as well as during FMLP challenge Ro 31-8220 (10 $\mu$M) inhibited primed $TXB_2$ release by > 80%. The results indicate that PTK activation is required for the generation of these inflammatory mediators by FMLP in AM. PKC activation appears to be required for LPS priming but not for transducing the FMLP signal; rather, PKC activation may modulate the signal by a negative feedback mechanism.**

**Key words:** Alveolar macrophage activation, Cell priming, Protein kinase C inhibitors, Protein tyrosine kinase inhibitors

### Protein tyrosine kinase but not protein kinase C inhibition blocks receptor induced alveolar macrophage activation

**K. Pollock and M. T. Withnall**[CA]

Rhône-Poulenc Rorer Ltd, Dagenham Research Centre, Rainham Road South, Dagenham, Essex RM10 7XS, UK

[CA] Corresponding Author

### Introduction

The alveolar macrophage (AM) is the most abundant cell in the airway lumen and may be an important contributor to the inflammation associated with pulmonary diseases such as asthma.[1,2] Isolated AM respond to a variety of immunological and non-immunological stimuli by generating both acute-phase and pro-inflammatory mediators. The acute-phase mediators include the spasmogens thromboxane $A_2$, leukotriene $C_4$ and platelet activating factor (PAF) as well as reactive oxygen species and lysosomal enzymes which can cause local tissue damage.[3] Among the pro-inflammatory mediators are PAF, leukotriene $B_4$ and a number of cytokines which are chemoattractant to mast cells, eosinophils and lymphocytes, all of which have been implicated in pulmonary inflammation.[1,2] In addition to being directly activated AM can be primed *in vitro* by agents including LPS[4] and gamma interferon[5] to give an exaggerated response to subsequent stimuli. AM primed *in vitro* may be comparable with AM from asthmatic subjects which have been shown to release greater amounts of many of the above mediators on stimulation than cells from non-asthmatic subjects.[6–9]

The biochemical mechanisms underlying priming are poorly understood but may involve amplification of signal transduction processes. For the chemotactic peptide FMLP three receptor-coupled signalling pathways have been identified: (i) G-protein linked phospholipase C (PLC) which generates inositol 1,4,5 trisphosphate and diacylglycerol (DAG) from phosphoinositides,[10] (ii) phospholipase D generation of DAG from phosphatidyl choline,[11] and (iii) activation of non-receptor PTK.[12,13] As PTK can activate PLC$\gamma$[14] to generate DAG, activation of PKC could represent a common end point for all of these pathways, although other mechanisms of PTK dependent cell activation clearly exist. Both PKC and PTK have been implicated in the priming process in macrophages; for instance in murine peritoneal macrophages LPS priming stimulated myristoylation and translocation of an intracellular substrate for PKC thus improving the efficiency of this signal transduction pathway,[15] whilst LPS priming in P388 D1 cells was shown to require protein synthesis and to be blocked by a PTK inhibitor.[16]

In order to investigate the importance of PTK and PKC enzyme systems in FMLP activation of AM we have studied the effects of the selective PTK inhibitor genistein[17] and the selective PKC inhibitor Ro 31-8220[18] on superoxide and $TXB_2$ (as a marker for arachidonate metabolism) generation in normal cells, and on $TXB_2$ generation in LPS primed cells. As a control measure the effects of these compounds on superoxide release induced by FMLP were compared with those on superoxide release induced

© 1993 Rapid Communications of Oxford Ltd by the phorbol ester PMA, which is known to act by stimulating PKC directly.

### Materials and Methods

Male Dunkin–Hartley guinea-pigs were purchased from David Hall, Burton-on-Trent, UK. Tissue culture reagents were obtained from Gibco; FMLP, phorbol myristate acetate (PMA), type III cytochrome c, LPS (*E. coli* 055:B5) and superoxide dismutase from Sigma, UK; and $TXB_2$ radioimmunoassay kits from Amersham, UK. Protein was determined after disruption of cells with 0.1% Triton X-100 using BioRad Coomassie blue reagent. Genistein was purchased from ICN-Flow whilst Ro 31-8220 was synthesized at Dagenham Research Centre. Compounds were initially dissolved in dimethylsulphoxide (DMSO) and diluted in Hank's balanced salt solution (HBSS) to a final DMSO concentration in cell incubates of $\leq$0.1% v/v.

*Preparation of AM:* Male guinea-pigs (400–600 g) were killed by intraperitoneal administration of sodium pentobarbitone, the trachea cannulated and bronchoalveolar lavage carried out with two 5 ml volumes of saline at 37°C. Lavage fluid was centrifuged at 300 × ***g*** for 8 min, cells resuspended in $Ca^{2+}$ and $Mg^{2+}$ free HBSS and pooled. The cell suspension was centrifuged again and cells resuspended at $10^6$ cells/ml in RPMI 1640 medium supplemented with 1% foetal calf serum, 50 U/ml penicillin and 50 $\mu$g/ml streptomycin. For superoxide experiments cells were plated out in 24-well dishes at $10^6$ cells/well; for $TXB_2$ experiments in 96-well dishes at $2 \times 10^5$ cells/well. After 2 h in an incubator (37°C, 5% $CO_2$) non-adherent cells and debris were removed by changing the medium. Adherent AM were left in the incubator for 18–20 h before use.

*Measurement of superoxide production:* Tissue culture medium was removed, AM washed once with HBSS then HBSS (0.5 ml) with or without inhibitors was added and the cells incubated at 37°C for 2 h. Buffer was then replaced by fresh HBSS (0.5 ml) containing $Fe^{3+}$ cytochrome c (55 $\mu$M), again with or without inhibitors. After 15 min at 37°C FMLP or PMA was added and the incubation continued for a further 30 min. Superoxide generation was determined from the difference in absorbance of the AM supernatants at 550 nm in comparison with supernatants from cells stimulated in the presence of superoxide dismutase (0.1 mg/ml).[19] Using this protocol the absorbance difference was directly proportional to superoxide concentration.

*Measurement of $TXB_2$ release:* After removal of tissue culture medium AM were washed once with HBSS then incubated for 2 h at 37°C in HBSS (0.2 ml) with or without inhibitors and with or without LPS (10 ng/ml) for cell priming. The buffer was replaced by fresh HBSS (0.2 ml) again with or without inhibitors, and the incubation continued for 15 min prior to adding FMLP for a further 15 min. Supernatants removed at appropriate times were stored at −20°C prior to quantification of $TXB_2$ by radioimmunoassay. Samples were diluted to fall within the assay standard curve; concentrations were calculated by interpolation using a Multi Calc software program (Wallac, Finland).

*Statistics:* Superoxide and $TXB_2$ concentrations were calculated as mean ± S.E.M. of triplicate or quadruplicate determinations within each experiment. Since the magnitude of stimulated release varied with different preparations of cells, in all cases the figures show values from one representative experiment which was conducted 2–4 times. $IC_{50}$ values and other data in the Results section are expressed as mean ±S.E.M. from the stated number of replicate experiments.

### Results

*Effects on superoxide generation:* Adherent guinea-pig AM prepared as described showed a very low basal generation of superoxide ($\leq$0.1 nmole/assay). Both FMLP and PMA (0.3–30 nM) caused concentration dependent stimulation of superoxide generation with $EC_{50}$ values of 1.2, 2.6 nM and 3.0, 5.4 nM, respectively, in two experiments and similar maximal stimulation (1–2 nmoles/assay). The effects of genistein and Ro 31-8220 were evaluated against sub-maximal (approx. $EC_{70}$) agonist concentrations (3 nM FMLP, 10 nM PMA) that gave similar responses (compare Figs 1A and 1B). Consistent with its activity as a PKC antagonist Ro 31-8220 inhibited PMA induced superoxide generation (Fig. 1A); in three experiments the $IC_{50}$ was 0.21 ± 0.10 $\mu$M. Whereas genistein at concentrations up to 100 $\mu$M had little effect on the PMA response it dose-dependently inhibited FMLP induced superoxide generation (Fig. 1B); in four experiments the $IC_{50}$ was 54 ± 9 $\mu$M. Ro 31-8220 at low concentrations (0.1–1 $\mu$M) had little or no inhibitory effect on the FMLP response whereas at higher concentrations (3 and 10 $\mu$M) it consistently enhanced the FMLP response (by 64 ± 16% at 10 $\mu$M; $n = 3$ experiments).

At the highest concentrations employed Ro 31-8220 and genistein did not reduce cell viability as assessed by trypan blue dye exclusion, viability being consistently $\geq$95%.

*Effects on $TXB_2$ release:* Pre-incubation of guinea-pig AM for 2 h with LPS (10 ng/ml) in itself caused some increase in $TXB_2$ release from a basal level of

FIG. 1. The effects of Ro 31-8220 and genistein on PMA (Panel A) or FMLP (Panel B) induced superoxide release from non-primed guinea-pig AM. AM ($10^6$/well) were incubated with or without inhibitors for 2.25 h at 37°C prior to addition of stimulus for 30 min. Superoxide was determined by superoxide dismutase inhibitable reduction of $Fe^{3+}$ cytochrome c. Mean ± S.E.M. ($n = 3$) from a single representative experiment.

FIG. 2. Demonstration of LPS priming of AM for $TXB_2$ release induced by FMLP. AM (2 × $10^5$/well) were incubated in HBSS or in HBSS containing LPS (10 ng/ml) for 2 h at 37°C then the medium replaced by fresh HBSS. After 15 min at 37°C FMLP was added for a further 15 min. $TXB_2$ was measured by RIA. Mean ± S.E.M. ($n = 4$) from a single representative experiment. ■, Non-primed; □, primed.

0.10 ± 0.01 to 1.0 ± 0.2 ng/10 μg AM protein ($n = 4$ experiments). However LPS potentiated markedly (primed) the response to FMLP over the full concentration range tested (0.3–30 nM) (Fig. 2). FMLP (3 nM), as a non-maximal stimulus, was used to study the effects of Ro 31-8220 and genistein on $TXB_2$ release.

When present during the 2 h pre-incubation, or 2 h LPS priming, stage and the subsequent challenge stage genistein (1–30 μM) dose-dependently inhibited FMLP stimulated $TXB_2$ release in both non-primed and primed AM with similar $IC_{50}$ values (17 and 18 μM, respectively, in the experiment shown in Fig. 3A). Under these conditions Ro 31-8220 (0.1–10 μM) did not inhibit FMLP induced $TXB_2$ release in non-primed AM but tended rather to potentiate release at higher concentrations (by >27% at 3 and 10 μM in the experiment shown) (Fig. 3B). The extent of potentiation varied between experiments but was a consistent observation. In LPS primed AM low concentrations of Ro 31-8220 (0.1–3 μM) had no effect on the FMLP response whereas Ro 31-8220 at 10 μM inhibited $TXB_2$ release by 80% relative to the LPS baseline level. Since this suggested that Ro 31-8220 had an inhibitory effect on a component of LPS priming, further studies were performed in which Ro 31-8220 (10 μM) was added to the AM only during selected stages of priming and/or activation (Fig. 4). Ro 31-8220 had no effect on the LPS primed response when present only during FMLP challenge, inhibited the response by approx. 50% when present only during priming, but had greatest effect, reducing FMLP induced $TXB_2$ release by 80 and 100% in relation to the LPS baseline value ($n = 2$ experiments), when present during both priming and challenge. The essential requirement for inhibition was thus that Ro 31-8220 was present during the LPS priming stage.

### Discussion

Ro 31-8220 and genistein were used in the present study as selective inhibitors of PKC and PTK, respectively. Ro 31-8220 is reported to have a 100-fold selectivity for PKC over protein kinase A (PKA) and a 1 000-fold selectivity over $Ca^{2+}$–calmodulin dependent kinase[18] and has been used by several groups as a selective PKC inhibitor in intact cells.[20–22] Genistein inhibits a number of PTKs but

FIG. 3. The effects of genistein (Panel A) or Ro 31-8220 (Panel B) on FMLP induced $TXB_2$ release from non-primed or LPS-primed AM. AM were primed with LPS (10 ng/ml) and stimulated with FMLP (3 nM) as described in the legend to Fig. 2. Genistein or Ro 31-8220 were added during both LPS priming and FMLP stimulation. Mean ± S.E.M. ($n = 4$) from a single representative experiment. □, Non-primed; ■, primed.

FIG. 4. Influence of the stage of LPS priming, or FMLP challenge at which Ro 31-8220 was added, on its ability to inhibit $TXB_2$ release. AM were primed with LPS (10 ng/ml) and stimulated with FMLP (3 nM) as described in the legend to Fig. 2. LPS + (Ro + FMLP): Ro 31-8220 added for 15 min prior to, and during FMLP stimulation, but not during priming; (Ro + LPS) + FMLP: Ro 31-8220 added during LPS priming but removed before FMLP stimulation; (Ro + LPS) + (Ro + FMLP): Ro 31-8220 added during LPS priming and for 15 min prior to, and during, FMLP stimulation. Mean ± S.E.M. ($n = 4$) from a single representative experiment.

shows very weak activity against serine and threonine kinases including PKC, PKA and phosphorylase kinase.[17] At the concentrations employed neither Ro 31-8220 nor genistein reduced AM viability, thus ruling out nonspecific cytotoxicity as an explanation of any observed effects of the compounds.

The finding that Ro 31-8220 inhibited PMA induced superoxide generation but not FMLP induced superoxide generation or $TXB_2$ release in non-primed AM indicates that PKC either does not transduce the FMLP signal or that it has feed back as well as feed forward effects on signal transduction. The fact that Ro 31-8220 consistently increased superoxide or $TXB_2$ release (albeit not markedly) rather than merely being without effect on release leads us to favour the latter explanation. There is increasing evidence from a number of cell types that PKC inhibition can increase cellular activation, apparently by blocking inhibitory effects of PKC on phospholipase C. For instance, Ro 31-8220 was shown to potentiate PAF stimulated $TXB_2$ release from platelets,[20] IgE stimulated histamine release from human basophils,[23] and zymosan induced phospholipase C activity in liver macrophages.[21] In addition, in the latter study the activation of phospholipase C by zymosan was enhanced by chronic pretreatment of the macrophages with PMA to down-regulate PKC. It is unlikely that a Ro 31-8220 insensitive isoform of PKC was involved in FMLP stimulated superoxide and $TXB_2$ production in the AM studied in the present work since Ro 31-8220 is not isozyme selective and, in addition, the compound inhibited the superoxide response to PMA.

In contrast to the complex activity of Ro 31-8220, the consistent inhibitory effects of genistein on FMLP responses in both non-primed and primed AM support only a proactive role for a PTK in FMLP stimulus–response coupling in AM, as has recently been reported for neutrophils[12,24] and for HL-60 cells.[13] Whether the pathways leading to superoxide or $TXB_2$ release are separately regulated by PTK or whether PTK action affects an early common event such as PLC$\gamma$ activation,[14] or PLD activation,[25] each of which has been demonstrated in other cell types, remains to be determined. Nonetheless, the ability of genistein to inhibit superoxide production and $TXB_2$ release almost completely emphasizes the key importance of PTK activation following FMLP receptor stimulation.

The observation that Ro 31-8220 was able to inhibit FMLP induced $TXB_2$ production in primed AM provided that the compound was present during the priming event is of interest in demonstrating that LPS induces priming by a process which is positively regulated by PKC. This does not appear surprising in view of other reports that LPS can activate PKC in macrophages[26] and that responses to LPS such as cytokine production are blocked by PKC inhibitors.[27] It does, however, differ from findings in P388 D1 macrophages where the non-selective PKC inhibitor H7 did not affect cell priming.[16] The fact that Ro 31-8220 present during FMLP challenge as well as during priming increased the inhibition of $TXB_2$ release contrasts with the potentiation of release seen in non-primed AM and probably reflects the complex interplay between feed forward and feed back effects of PKC on mediator release in these cells.

In summary, using selective enzyme inhibitors it was found that activation of PTK is a key event in FMLP generation of superoxide and $TXB_2$ in guinea-pig AM whereas PKC either is not activated, or is activated but has counter-balancing feed back and feed forward effects on mediator release in non-primed cells. On the other hand activation of PKC appears to be required for LPS priming of these cells for enhanced $TXB_2$ release. Thus both PTK and PKC inhibitors could conceivably be effective in reducing the release of these mediators from macrophages in the airways *in vivo* under conditions where priming has occurred during an inflammatory response.

### References

1. Burke LA, Wilkinson JR, Howell CJ, Lee TH. Interactions of macrophages and monocytes with granulocytes in asthma. *Eur Respir J* 1991; **4** (Suppl 13): 85s–90s.
2. Fuller RW. Pharmacological regulation of airway macrophage function. *Clin Exp Allergy* 1991; **21**: 651–654.
3. Fels AO, Cohn ZA. The alveolar macrophage. *J Appl Physiol* 1986; **60**: 353–369.
4. Aderem AA, Cohen DS, Wright SD, Cohn ZA. Bacterial lipopolysaccharides prime macrophages for enhanced release of arachidonic acid metabolites. *J Exp Med* 1986; **164**: 165–179.
5. Eden E, Turino GM. Interleukin 1 secretion by human alveolar macrophages stimulated with endotoxin is augmented by recombinant immune (gamma) interferon. *Am Rev Respir Dis* 1986; **133**: 455–460.
6. Cluzel M, Damon M, Le Doucen C, Michel FB, Crastes de Poulet A, Godard P. Enhanced alveolar cell luminol-dependent chemiluminescence in asthma. *J Allergy Clin Immunol* 1987; **80**: 195–201.
7. Joseph M, Tonnel AB, Torpier G, Capron A, Arnoux B, Benveniste J. The involvement of IgE in the secretory processes of alveolar macrophages from asthmatic patients. *J Clin Invest* 1983; **71**: 221–230.
8. Godard P, Chantreuil J, Damon M *et al.* Functional assessment of alveolar macrophages: comparison of cells from asthmatic and normal subjects. *J Allergy Clin Immunol* 1982; **70**: 88–93.
9. Howell CJ, Pujol JL, Crea AEG *et al.* Identification of an alveolar macrophage derived activity in bronchial asthma which enhances leukotriene $C_4$ generation by human eosinophils stimulated by ionophore (A23187) as granulocyte-macrophage-colony-stimulating factor (GM-CSF). *Am Rev Respir Dis* 1989; **140**: 1340–1347.
10. Hamilton TH, Adams DO. Molecular mechanisms of signal transduction in macrophages. *Immunol Today* 1987; **8**: 151–158.
11. Thompson NT, Tateson JE, Randall RW, Spacey GD, Bonser RW, Garland LG. The temporal relationship between phospholipase activation, diradylglycerol formation and superoxide production in the human neutrophil. *Biochem J* 1990; **271**: 209–213.
12. Kusunoki T, Higashi H, Hosai S *et al.* Tyrosine phosphorylation and its possible role in superoxide production by human neutrophils stimulated with FMLP and IgG. *Biochem Biophys Res Commun* 1992; **183**: 789–796.
13. Offermanns S, Seifert R, Metzger JW *et al.* Lipopeptides are effective stimulators of tyrosine phosphorylation in human myeloid cells. *Biochem J* 1992; **282**: 551–557.
14. Klausner RD, Samelson LE. T cell antigen receptor activation pathways: the tyrosine kinase connection. *Cell* 1991; **64**: 875–878.
15. Aderem AA, Albert KA, Keum MM, Wang JK, Greengard P, Cohn ZA. Stimulus-dependent myristoylation of a major substrate for protein kinase C. *Nature* 1988; **332**: 362–364.
16. Glaser KB, Asmis R, Dennis EA. Bacterial lipopolysaccharide priming of P388 D1 macrophage-like cells for enhanced arachidonic acid metabolism. *J Biol Chem* 1990; **265**: 8658–8664.
17. Akiyama T, Ishida J, Nakagawa S. Genistein, a specific inhibitor of tyrosine-specific protein kinases. *J Biol Chem* 1987; **262**: 5592–5595.
18. Twomey B, Muid RE, Nixon JS, Sedgwick AD, Wilkinson SE, Dale MM. The effect of new potent selective inhibitors of protein kinase C on the neutrophil respiratory burst. *Biochem Biophys Res Commun* 1990; **171**: 1087–1092.
19. Metcalf JA, Gallin JI, Nauseef WM, Root RK. *Laboratory Manual of Neutrophil Function.* New York: Raven Press, 1986; 109–114.
20. Murphy CT, Westwick J. Selective inhibition of protein kinase C. Effect on platelet-activating-factor-induced platelet functional responses. *Biochem J* 1992; **283**: 159–164.
21. Dieter P, Fitzke E. Ro 31-8220 and Ro 31-7549 show improved selectivity for protein kinase C over staurosporine in macrophages. *Biochem Biophys Res Commun* 1991; **181**: 396–401.
22. Walker TR, Watson SP. Effect of Ro 31-8220, a novel PKC inhibitor, on human platelet phosphorylation and secretion. *Br J Pharmacol* 1991; **104**: 88P.
23. Amon U, von Stebut E, Dietz KR, Bauer FW, Wolff HH. Pharmacological studies on the role of protein kinase C in signal transduction. *Int Arch Allergy Immunol* 1992; **98**: 349–354.
24. Gomez-Cambronero J, Huang CK, Bonak VA *et al.* Tyrosine phosphorylation in human neutrophil. *Biochem Biophys Res Commun* 1989; **162**: 1478–1485.
25. Uings IJ, Thompson NT, Randall RW *et al.* Tyrosine phosphorylation is involved in receptor coupling to phospholipase D but not phospholipase C in the human neutrophil. *Biochem J* 1992; **281**: 597–600.
26. Wightman PD, Raetz CRH. The activation of protein kinase C by biologically active moieties of lipopolysaccharide. *J Biol Chem* 1984; **259**: 10048–10052.
27. Coffey RG, Weakland LL, Alberts VA. Paradoxical stimulation and inhibition by protein kinase C modulating agents of lipopolysaccharide evoked production of tumour necrosis factor in human monocytes. *Immunology* 1992; **76**: 48–54.

ACKNOWLEDGEMENT. We thank Emma Burke for secretarial assistance in the preparation of this manuscript.

**Received 16 June 1993;**
**accepted in revised form 27 July 1993**
